# Long-Term Results of Neurological Outcome, Quality of Life, and Cosmetic Outcome After Cranioplastic Surgery: A Single Center Study of 202 Patients

**DOI:** 10.3389/fneur.2021.702339

**Published:** 2021-07-20

**Authors:** Henrik Giese, Jennifer Antritter, Andreas Unterberg, Christopher Beynon

**Affiliations:** Department of Neurosurgery, University Hospital Heidelberg, Heidelberg, Germany

**Keywords:** cranioplasty, decompressive craniecotmy, quality of life, cosmetic result, neurorehabilitation

## Abstract

**Objective:** An increased interest in the surgical procedures of decompressive craniectomy (DC) and subsequent cranioplasty (CP) has emerged during the last decades with specific focus on mortality and complication rates. The aim of the present study was to evaluate long-term neurological and cosmetic outcomes as well as Quality of Life (QoL) after CP surgery.

**Methods:** We retrospectively reviewed the medical records of CP patients treated at our institution between 2004 and 2014 and performed a follow-up examination, with evaluation of neurological outcome using the modified Rankin Scale (mRS) and the Glasgow outcome scale (GOS), QoL (SF-36 and EQ-5D-3L). Furthermore, the cosmetic results after CP were analyzed.

**Results:** A total of 202 CP-patients were included in the present study. The main indications for DC and subsequent CP were space-occupying cerebral ischemia (32%), traumatic brain injury (TBI, 26%), intracerebral or subarachnoid hemorrhage (32%) and infection (10%). During a mean follow-up period of 91.9 months 46/42.6% of patients had a favorable neurological outcome (mRS ≤ 3/GOS ≥ 4). Patients with ischemia had a significant worse outcome (mRS 4.3 ± 1.5) compared with patients after TBI (3.1 ± 2.3) and infectious diseases requiring CP (2.4 ± 2.3). The QoL analysis showed that <1/3rd of patients (31.2%) had a good QoL (SF-36) with a mean EQ-5D-VAS of 59 ± 26. Statistical analysis confirmed a significant worse QoL of ischemia patients compared to other groups whereas multivariate regression analysis showed no other factors which may had an impact on the QoL. The majority (86.5%) of patients were satisfied with the cosmetic result after CP and regression analysis showed no significant factors associated with unfavorable outcomes.

**Conclusion:** Long-term outcome and QoL after CP were significantly influenced by the medical condition requiring DC. Early detection and evaluation of QoL after CP may improve the patient's outcome due to an immediate initiation of targeted therapies (e.g., occupational- or physiotherapy).

## Introduction

Cranioplasty (CP) after (decompressive) craniectomy (DC) is an essential surgical procedure in order to cover the originated skull defect, to protect the patient's brain from outside forces and to re-integrate patients into “normal” life. Nevertheless, CP is often associated with considerable complication rates of up to 36% (1–6). Several studies have analyzed potential risk factors for complications (4, 7–14).

The role of DC in the treatment of stroke and traumatic brain injury (TBI) is a matter of ongoing debate. The potential to reduce mortality has to be balanced against the increased risk of major disability. The neurological outcome is primarily dependent on the initial condition requiring surgical DC but also on other factors such as age, time of DC, experience of treating centers, and subsequent problems after CP (15). Neurological rehabilitation and especially quality of life (QoL) in respective patients have gained increased attention. It has been well-established that the potential for neurological improvement after CP surgery is present especially during the first month after DC (16). Nevertheless, only limited data are available on the neurological long-term outcome and QoL after CP-surgery. Only one study has investigated the quality of life (QoL) after CP so far and it has demonstrated that QoL improved after a period of 24 months (17). Patients with favorable outcome after DC and CP desire to return to normal life as quickly as possible. Cosmetic stigmata such as conspicuous scaring or retraction/bulging of the scalp can have a significant impact on individuals. These abnormalities can lead to daily confrontation of patients with their disease and subsequent negative effects on their activities.

The aim of this study was to evaluate the long-term neurological outcome after CP and particularly the QoL and cosmetic results after CP. In addition, possible differences in the various diseases (ischemia, TBI, intracerebral and subarachnoid hemorrhage, infection) were analyzed.

## Methods

### Patient Characteristics and Study Design

The study protocol was approved by the local standing committee on ethical practice (Ethics Committee of University of Heidelberg). Between 2004 and 2014 we performed a total of 498 CPs in 382 patients at our institution. Patients were included if they required decompressive craniectomy (DC) as part of their neurocritical management (e.g., in case of a malignant space occupying stroke or severe TBI) or craniectomy due to infectious diseases (e.g., encephalitis, osteomyelitis). All patients lost to follow-up, underage or that rejected study participation were excluded from further analysis. In addition, all patients without a recent CP (at the time of our survey) or patients with CP after tumor removal (e.g., osseous meningiomas) were excluded as well. Medical records were retrospectively reviewed and patient data regarding demographic information, specific risk factors, images, details of the surgery as well as in-hospital course were analyzed. All patients were contacted by phone, letter, e-mail or their primary care physician. Before, all participants were provided a verbal and written informed consent. After study agreement, the neurological status [modified Ranking Scale (mRS), Glasgow Outcome Scale (GOS)] was evaluated by an interview. A favorable neurological outcome was defined as mRS score ≤ 3 and GOS score ≥ 4. Furthermore, patients were asked to complete further questionnaires for evaluation of QoL as well as cosmetic results.

### Quality of Life and Cosmetic Outcome

The concept of “Quality of Life” describes the conditions of human's life, considering several factors such as health, material, family, professional and other social factors. In order to evaluate and quantify the QoL of different patients in this study, we used the Short Form 36 Health Survey (SF-36) and the EQ-5D-3L questionnaire. The SF-36 was developed by the RAND Corporation and consists of eight scaled scores, which are the weighted sums of the questions in their section. Each scale is directly transformed into a 0–100 scale on the assumption that each question weighs equally. A lower score indicates a higher degree of disability (18, 19). EQ-5D-3L is a standardized instrument developed by the EuroQol Group as a measure of health-related QoL (http://www.euroqol.org). The score consists of a descriptive system (five dimensions) and the EQ-VAS (Visual Analog Scale) (20). Furthermore, the five dimensions are summarized in the EQ-5D-index which also reflects the status of health like the VAS. For evaluation of the cosmetic result, predefined cosmetic scores are not available. Therefore, we created a special questionnaire which encompasses the cosmetic results of the skull, functional problems (e.g., chewing restrictions), scars as well as subjective problems (e.g., paresthesia, pain etc.). Questionnaires were completed by patients and/or their relatives.

### Classification of CP Patients

For descriptive and statistical analysis patients were subdivided into four groups depending on their pathology/indication for DC. Patients with DC due to infratentorial pathology were excluded from the analysis.

#### Ischemia

Patients with DC due to malignant, supratentorial, cerebral infarction/ischemia (in the majority of cases due to middle cerebral or internal carotid artery occlusion).

#### TBI

Patients with supratentorial traumatic hemorrhage (epidural, subdural, intracerebral) and subsequent DC due to intracranial hypertension.

#### Intracerebral/Subarachnoid Hemorrhage

All patients with aneurysmal subarachnoid hemorrhage, spontaneous intracerebral hemorrhage or hemorrhage due to cerebral sinus venous thrombosis with intracranial hypertension.

#### Infection

Patients with intracranial hypertension due to (bacterial) encephalitis. Furthermore, all patients with secondary osteomyelitis after neurosurgical intervention were included.

### Statistical Analysis

Data were collected in an Excel database followed by a statistical analysis using a standard SPSS software package (Version 25, IBM Corp.). Absolute and relative frequencies are presented as means and standard deviation. A critical difference of 5% (*p* = 0.05) was assumed to be statistically significant. Patient survival rates were analyzed by using Kaplan Meier survival analysis, followed by log rank test. *T*-tests were used to compare the neurological outcome after initial CP and long-term follow up. Differences in cosmetic outcome between different materials were also assessed by student's *t*-test. SF-36 data was computed using the analyzing-package for SPSS. ANOVA (variance analysis) was used to identify significant differences in outcome between different indication-groups regarding SF-36 and EQ-5D-3L data. *T*-tests with Bonferroni correction were conducted as *post-hoc* tests. Multivariate regression analysis was performed for evaluation of factors which may influence the QoL (SF-36).

## Results

### Patient Cohort

**Figure 1:**
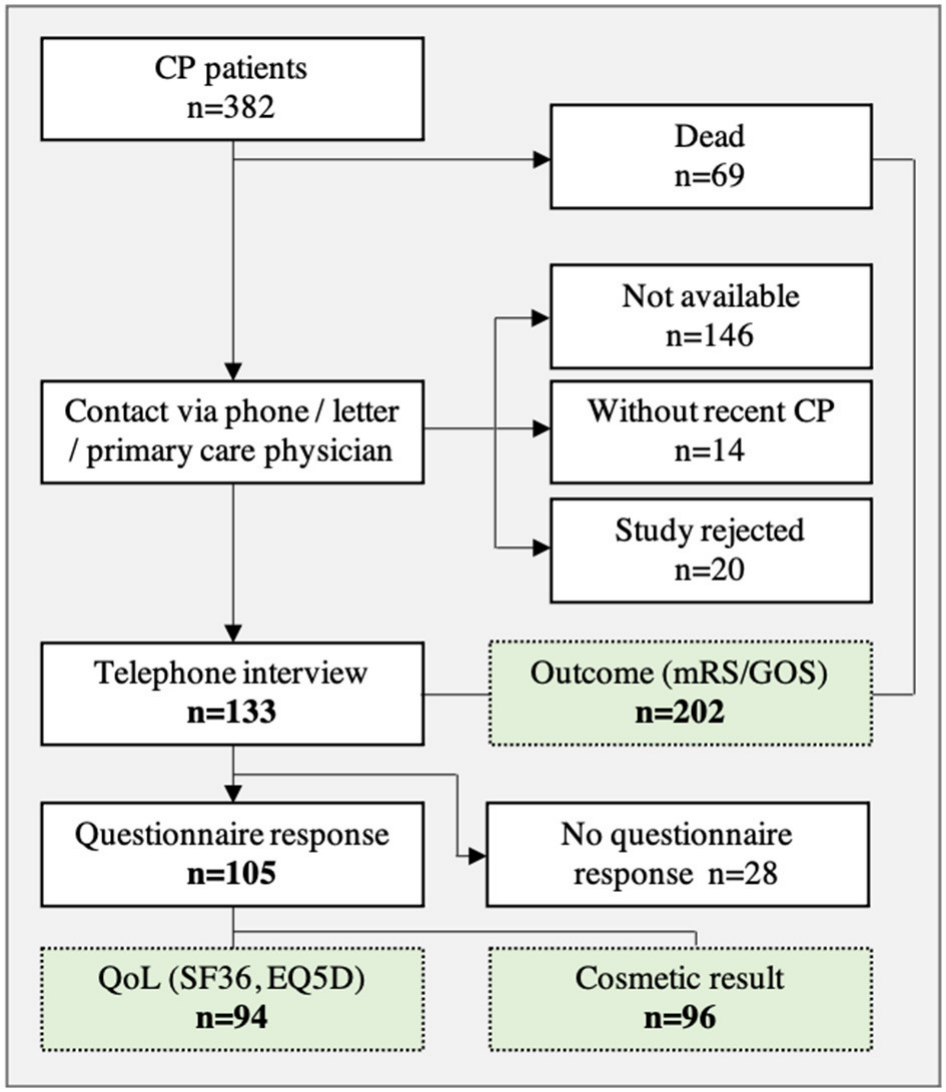
Patient collective after CP surgery and follow-up analysis (green).

In a first step, a total of 382 patients were included in the retrospective analysis (Figure 1). Thereafter, 180 patients were excluded from analysis due to study rejection or loss to follow-up as well as patients without recent CP (at time of survey). In addition, 69 patients died during the follow-up period, but were included in the neurological outcome analysis (mRS, GOS). A total of 133 patients agreed to participate in the telephone interview and neurological assessment was carried out in these patients.

The most common underlying pathologies for performing a craniectomy prior to CP were ischemia (32%), TBI (26%), and ICH/aSAH (32%). In Table 1 patient population (age, gender) and patient-specific risk factors, subdivided in indication groups, are presented in detail. Overall, the mean time between DC and initial CP was 158 ± 133 days. The majority of patients (*n* = 103) were treated with autologous CP whereas the remaining patients (*n* = 30) received an alloplastic implant. In 48 cases revision surgery was necessary due to CP complications like bone flap osteolysis, infection or wound healing disorders.

**Table 1:** Patient population, specific risk factors, and neurological outcome after CP.

	**Ischemia**	**TBI**	**ICH/aSAH**	**Infection**
**Overall patient (*****n*** **=** **202)**	*n* = 66, 32%	*n* = 52, 26%	*n* = 64, 32%	*n* = 20, 10%
Gender (f/m) %	45/55%	37/63%	61/39%	45/55%
Age at DC (mean ± SD)	53 ± 12 y	37 ± 19 y	47 ± 15 y	47 ± 17 y
Mortality rate during follow-up (n, %)	36%	27%	41%	25%
**Risk factors at DC**
Arterial hypertension	85%	29%	54%	40%
Diabetes mellitus	31%	6%	12%	5%
Other cardiovascular risk factors	63%	18%	25%	20%
Current smoker	42%	25%	31%	35%
Multidrug-resistant bacteria	11%	25%	18%	90%
**DC details**
Mean size (a × b in cm)	13.6 × 9.3	12.4 × 8.4	13.0 × 8.9	7.6 × 6.5
DC side (right/left in %)	55/45	49/51	55/45	50/50
**Neurological outcome (follow up)**
mRS (mean ± SD)	4.3 ± 1.6	3.1 ± 2.3	3.9 ± 2	2.4 ± 2.3
GOS (mean ± SD)	2.5 ± 1.3	3.3 ± 1.7	2.9 ± 1.7	3.8 ± 1.6
**Interview patients (*****n*** **=** **133)**	*n* = 42, 31%	*n* = 38, 29%	*n* = 38, 29%	*n* = 15, 11%
Gender (f/m) %	54/46%	46/54%	58/42%	60/40%
Age at DC (mean ± SD)	51 ± 12 y	30 ± 15 y	44 ± 14 y	43 ± 18 y

*Patients were subdivided into the four main indication groups as well as in overall and interview patients (n, number; %, proportion; y, years; TBI, Traumatic Brain Injury; ICH/aSAH, Intracerebral/subarachnoid hemorrhage; DC, decompressive craniectomy; mRS, modified Ranking Scale; GOS, Glasgow Outcome Scale*.

Questionnaires (SF36, EQ-5D-3L, cosmetic results) were sent to 133 patients and a response was received from 105 patients. After reviewing questionnaires, 96 complete sets were available for the analysis of cosmetic results and 94 sets were available for the analysis of QoL (Figure 1).

### Underlying Condition Requiring Initial Surgical Treatment

All patients (*n* = 202) were subdivided into four indication groups for the analysis of risk-factors (Table 1). Factors were retrospectively analyzed at the time of craniectomy. CP patients after ischemia showed a higher risk profile then all other groups. In detail, ischemia patients had a significantly higher age (*p* = 0.001), showed a higher rate of diabetes (*p* = 0.008), and arterial hypertension (*p* = 0.001) compared to TBI patients. Furthermore, ischemia patients had significantly more other cardiovascular risk factors than all other groups (*p* < 0.05). The extent of DC showed also significant differences (*p* < 0.05) between the emergency groups (ischemia, TBI, and ICH/aSAH) and the infection group (Table 1).

### Neurological Outcome and Survival Rate

**Figure 2:**
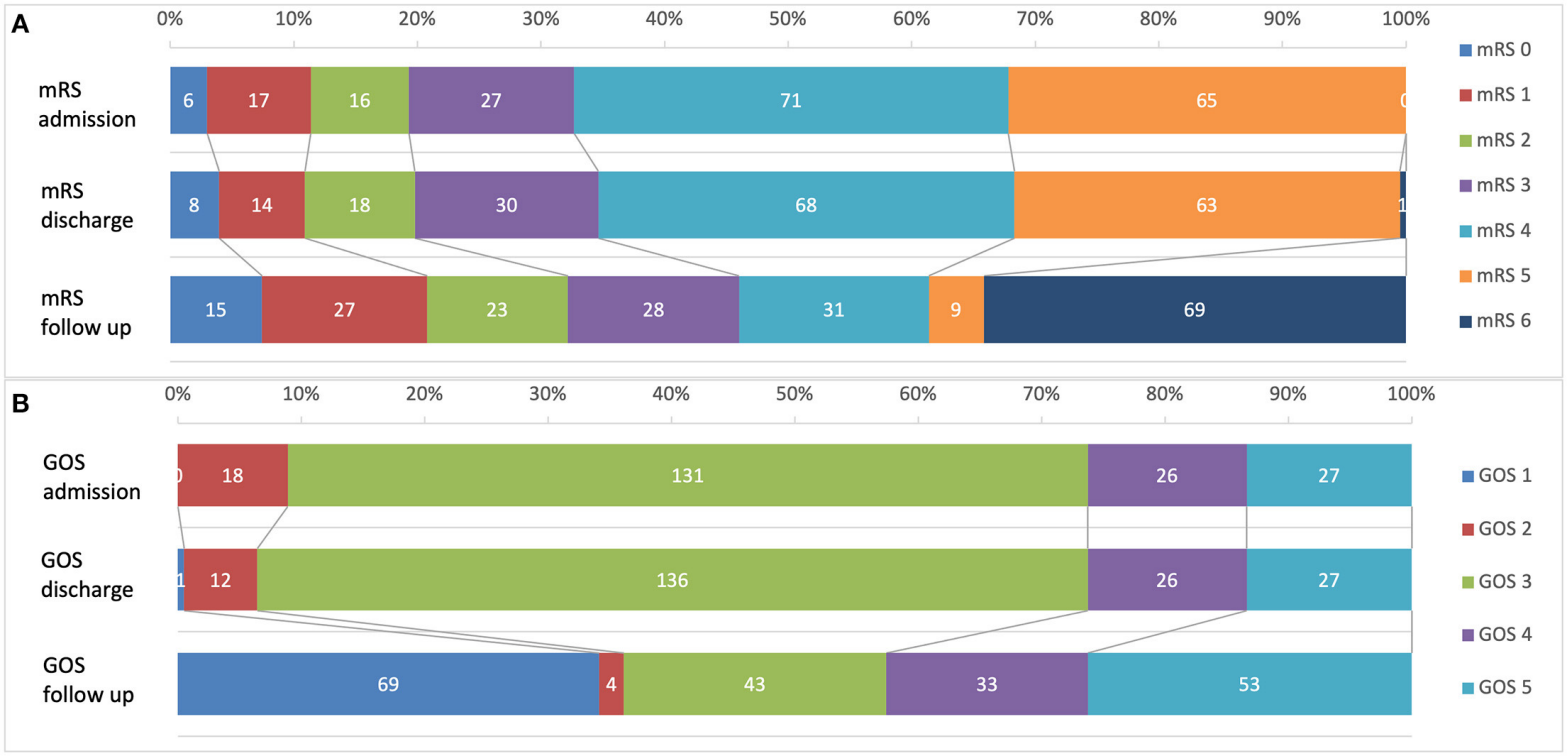
Neurological outcome of 202 CP patients before surgery, after surgery and during a mean follow-up of 91.9 month. **(A)** Modified Ranking scale (mRS); **(B)** Glasgow Outcome Scale (GOS).

A total of 202 patients were analyzed. Figure 2 illustrates mRS and GOS at admission and discharge during initial CP surgery and after a mean follow up period of 91.9 months. About half of patients (46/42.6%) showed a favorable neurological outcome (mRS ≤ 3/GOS ≥ 4) during the long-term observation. An unfavorable neurological outcome (mRS 4 and 5) was observed in 19.8% of patients and 34.1% of patients died during the follow up period from other causes than CP surgery. The 30-day mortality rate after CP was 0.49% (one patient died due to an acute cardiac event in hospital after CP surgery).

Short-term analysis of mRS/GOS at time of admission for CP-surgery and before discharge showed no significant (*p* = 0.1) differences of neurological outcome (Figure 2). Nevertheless, a significant increase of favorable neurological outcome was observed between initial scores before/after CP and follow up examination (*p* = 0.000/0.001).

Statistical analysis showed differences for the CP indication groups (Table 1). Patients after ischemia had significantly higher mRS (4.3 ± 1.5) than patients after TBI (3.1 ± 2.3, *p* = 0.023) or infection CP (2.4 ± 2.3, *p* = 0.006).

**Figure 3:**
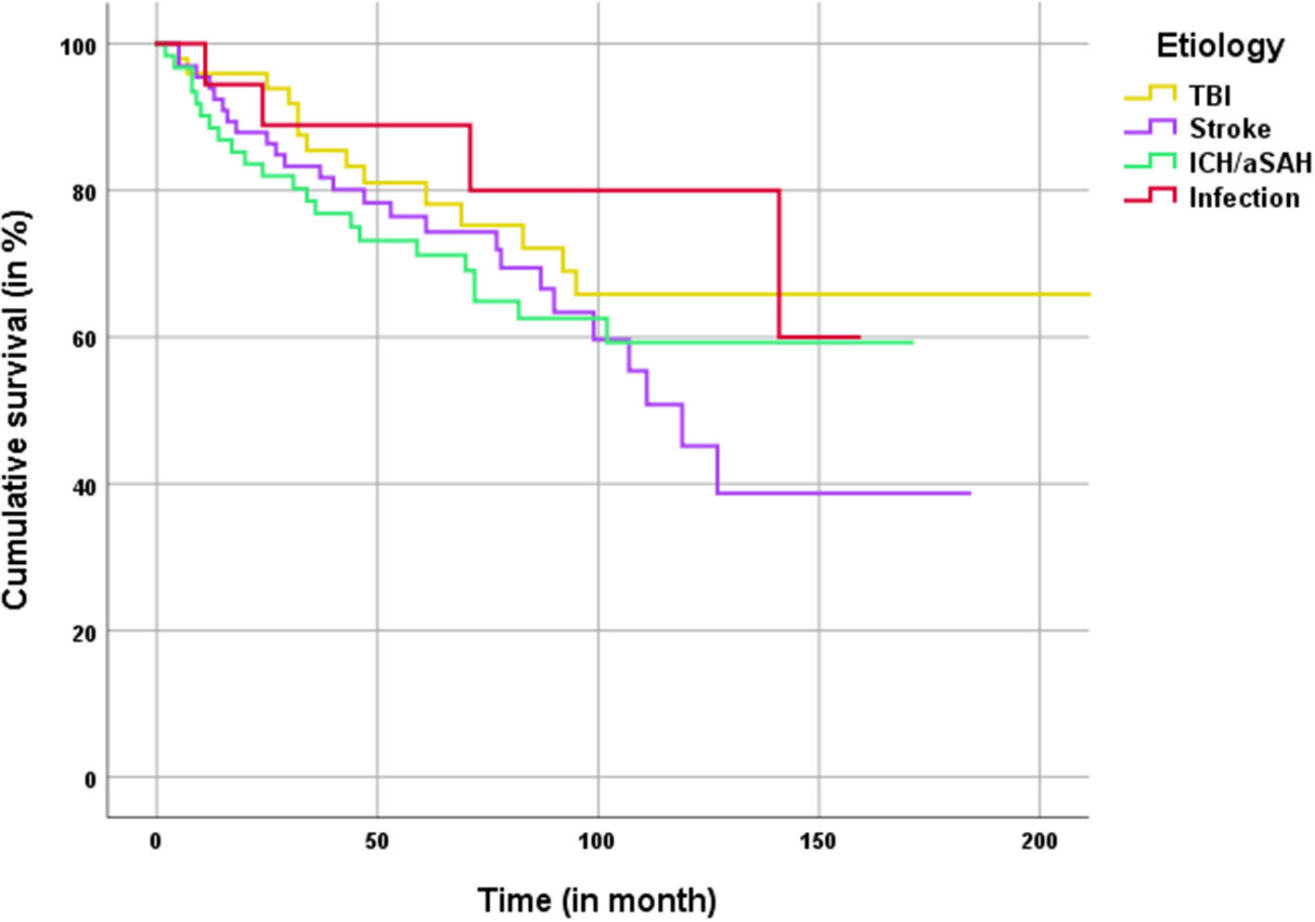
Kaplan–Meier analysis for patient survival classified by the main etiology for craniectomy.

Kaplan–Meier analysis for overall patient survival after DC and subsequent CP showed a mean estimated survival of 169 ± 9.1 month. No significant differences in patient survival rates were observed between different underlying conditions requiring CP (Figure 3). In the long-term (>100 months) the Kaplan–Meier-graph of TBI and ICH/aSAH patients showed a favorable trend compared to patients with CP after ischemia.

### Quality of Life

Data on evaluation of QoL were available in 94 patients. All results of the SF-36 are presented in Table 2, divided in overall results and compared with a healthy control group (normalized sample). In our CP collective, <1/3rd of patients had a good QoL regarding the physical (31.7%) and mental summary score (25%). About half of patients had a poor QoL in comparison to the healthy population. In 19 patients, a full analysis was not possible due to incomplete questionnaires (separate column in Table 2). The variance analysis (ANOVA) revealed a significant difference between the indication groups in the sub-categories physical functioning (*p* = 0.001), role-physical (*p* = 0.044), and vitality (*p* = 0.008) as well as the physical summary score (*p* = 0.001). In addition, *post-hoc* analysis showed significant differences in physical functioning (*p* = 0.016) and physical component summary (*p* = 0.015) between ischemia patients and all other groups. In conclusion, patients with CP after ischemia accordingly showed a worse physical status than those of other indication-groups. However, no significant differences were observed in other SF-36-subcategories.

**Table 2:** Results of SF-36 compared in overall and QoL in relation to comparable data set of a healthy control group (*n*, number; %, proportion).

	**Overall**	**Compared to healthy control group**		
	**Mean ± SD**	**Good QoL**	**Poor QoL**	**N.A**.
		***n* (%)**	***n* (%)**	***n* (%)**
Physical functioning (PF)	51.0 ± 38.7	47 (48.9)	46 (47.9)	3 (3.1)
Role-physical (RP)	50.3 ± 45.4	44 (45.8)	38 (39.6)	14 (14.6)
Bodily pain (BP)	69.3 ± 30.8	28 (29.2)	60 (62.5)	8 (8.3)
General health (GH)	58.3 ± 23.9	36 (37.5)	57 (59.4)	3 (3.1)
Vitality (VT)	43.9 ± 21.8	45 (46.9)	48 (50)	3 (3.1)
Social functioning (SF)	61.9 ± 34.5	46 (47.9)	47 (49)	3 (3.1)
Role-emotional (RE)	57.7 ± 47.9	32 (33.3)	48 (50)	16 (16.7)
Mental health (MH)	63.1 ± 21.5	33 (34.4)	59 (61.4)	4 (4.2)
Physical component summary (PCS)	42.3 ± 12.1	30 (31.2)	47 (49)	19 (19.8)
Mental component summary (MCS)	44.9 ± 12.7	24 (25)	53 (55.2)	19 (19.8)

The results of the EQ-5D-3L are described as a “Health Profile” and were separated according to similar indication groups (Table 3). Furthermore, the EQ-5D-index and the VAS were analyzed. Both values represent the patients' state of health. Normal range (best to worse) of the EQ-5D-index was 0.999 to −0.205 and for the VAS from 0 to 100. The overall (all indication groups) mean EQ-5D-index was 0.65 ± 0.34. Again, significant differences were observed in ischemia patients (0.44 ± 0.38) compared to other groups (e.g., EQ5D-index 0.82 ± 0.26 in CP patients after infection). The variance analysis (ANOVA) and *post-hoc* test also confirmed a significant worse QoL of ischemia patients compared to patients with infection and TBI (*p* = 0.015). The analysis of the VAS showed a mean VAS for all indication groups of 59 ± 26. The best QoL was observed in patients after infection CP (79.5 ± 21.1) followed by TBI-CP (67.6 ± 28.2) and patients of the ICH/aSAH group (52.9 ± 27.1). Again, worse results were observed in patients after ischemia CP (48.8 ± 21.8). Nevertheless, a statistically significant difference was found in the infection group (*p* = 0.003) compared to ischemia patients.

**Table 3:** Results of EQ-5D-3L for all indication groups.

		**TBI (*n* = 24)**	**Ischemia (*n* = 28)**	**ICH/aSAH (*n* = 25)**	**Infection (*n* = 13)**
Mobility	1	16 (66.7)	7 (25.0)	12 (44.4)	11 (84.6)
	2	7 (29.2)	14 (50.0)	12 (44.4)	2 (15.4)
	3	1 (4.2)	7 (25.0)	3 (11.1)	–
Self-care	1	18 (75.0)	4 (13.8)	17 (63.0)	12 (92.3)
	2	4 (16.7)	13 (44.8)	6 (22.2)	1 (7.7)
	3	2 (8.3)	12 (41.4)	4 (14.8)	–
Usual activities	1	13 (54.2)	5 (17.2)	10 (37.0)	6 (46.2)
	2	8 (33.3)	12 (41.4)	8 (29.6)	6 (46.2)
	3	3 (12.5)	12 (41.4)	9 (33.3)	1 (7.7)
Pain/discomfort	1	11 (45.8)	8 (27.6)	10 (38.5)	7 (53.8)
	2	11 (45.8)	16 (55.2)	15 (57.7)	4 (30.8)
	3	2 (8.3)	5 (17.2)	1 (3.8)	2 (15.4)
Anxiety/depression	1	12 (50.0)	5 (17.2)	10 (40.0)	9 (69.2)
	2	11 (45.8)	18 (62.1)	13 (52.0)	4 (30.8)
	3	1 (4.2)	6 (20.7)	2 (8.0)	–
EQ5D-index (Mean ± SD)		0.79 ± 0.28	0.44 ± 0.38	0.69 ± 0.29	0.82 ± 0.26

Finally, a multivariate regression analysis was performed for evaluation of factors which may influence the QoL (SF-36). No significant differences between groups were observed in post-operative complications, patient's age or CSF-shunt dependency.

### Cosmetic Outcome

Analysis of the cosmetic outcome was possible in a total of 96 patients (Table 4). The majority (86.5%) of patients were satisfied with the cosmetic result. Only 13.5% reported a poor cosmetic result. A detailed regression analysis showed no significant factors with a negative impact. Functional limitations were observed in 25% of patients, most commonly due to asymmetrical frown or chewing restrictions. Two thirds of patients (*n* = 59; 61.5%) had temporal muscle atrophy and about one third of patients suffered from local pain (*n* = 21; 21.8%), paresthesia (*n* = 37; 38.5%) or temperature discomfort (*n* = 20; 20.8%). We also compared the results of patient specific implants (PSI) and non-PSI CP. Only the “subjective feeling of CP loosening” showed a significant difference between the different groups (*p* = 0.032) with increased “subjective loosening” in the PSI-group. In conclusion, no significant differences in the cosmetic results between PSI- and autologous CP were observed.

**Table 4:** Cosmetic result after CP divided in patient specific implant (PSI) and non-PSI (*n*, number; %, proportion).

	**Overall**	**PSI**	**Non-PSI**	***p***
	***n* = 96**	***n* = 39**	***n* = 57**	
	***n* (%)**	***n* (%)**	***n* (%)**	
**Cosmetic result**				
Very good	14 (14.6)	7 (7.3)	7 (7.3)	0.223
Good	38 (39.6)	13 (13.5)	25 (26.0)	0.177
Satisfactory	29 (30.2)	10 (10.4)	19 (19.8)	0.197
Poor	13 (13.5)	7 (7.3)	6 (6.2)	0.053
**Scarring**				
Inconspicuous	47 (48.9)	17 (17.7)	30 (31.2)	0.244
Conspicuous	42 (43.8)	19 (19.8)	23 (23.9)	0.211
Bulging	7 (7.3)	3 (3.1)	4 (4.2)	0.451
**Unevenness of the CP area**	66 (68.7)	28 (29.2)	38 (29.6)	0.343
Visual	6 (6.3)	4 (4.2)	2 (2.1)	0.096
Palpable	17 (17.9)	7 (7.3)	10 (10.4)	0.495
Both	43 (45.3)	18 (18.7)	25 (26.0)	0.394
**Retraction/unevenness of the scalp**	88 (91.6)	36 (37.5)	52 (54.2)	0.426
Visual	3 (3.1)	1 (1.0)	2 (2.1)	0.398
Palpable	42 (43.8)	20 (20.8)	22 (22.9)	0.111
Both	43 (44.8)	15 (15.6)	28 (29.2)	0.154
**Functional limitation**	24 (25)	8 (8.3)	16 (16.7)	0.195
Problems during chewing of solid food	8 8.3)	4 (4.2)	4 (4.2)	0.304
Pain during chewing	2 (2.0)	1 (1.0)	1 (1.0)	0.397
Problems with eyelid closure	5 (5.2)	1 (1.0)	4 (4.2)	0.167
Asymmetrical frown	17 (17.7)	4 (4.2)	13 (13.5)	0.055
**Temporalis muscle atrophy**	59 (61.5)	26 (60.5)	35 (58.3)	0.83
**Pain/paresthesia**				
Pain of CP area	21 (21.8)	10 (10.4)	11 (11.4)	0.417
Paresthesia of CP area	37 (38.5)	17 (17.7)	20 (20.8)	0.154
Temperature paresthesia	20 (20.8)	10 (10.4)	10 (10.4)	0.389
Subjective feeling of CP loosening	14 (14.6)	9 (9.4)	5 (5.2)	0.032

## Discussion

Here, we present a study on the neurological long-term outcome after cranioplasty surgery. A total of 202 patients were analyzed with a mean follow up period of ~8 years. Significant differences in patient outcome and QoL were observed for the four main CP indications.

### Neurological Outcome

About half of patients had a favorable neurological outcome with an mRS of ≤ 3 or GOS ≥4. To the best of our knowledge, long-term results for CP patients have not been reported yet. Only a few short- or mid-term results up to 30 months in DC patients following TBI have been reported so far (21–24). The rate of good recovery ranged between 36 and 64.8% (21, 22, 24). In a similar heterogenous patient population (*n* = 204), a favorable outcome 1 year after DC was observed in 34% of patients and furthermore, a high variety of factors that had an impact on outcome parameters was reported (25). The results of our study are well-corresponding to these findings as patients with TBI or infectious conditions had significantly better outcomes than patients with ischemia.

The present study also demonstrates that a significant neurological improvement in the long-term course after CP can be observed in all patients requiring CP. An improvement of neurological function (mRS) before and after CP was seen only in four patients, probably due to the short observation period between admission and discharge after initial CP. A systematic review and meta-analysis of 528 patients confirmed a significant neurological improvement after CP (mean follow up 3–180 days) (16). The authors included seven CP studies with similar pre- and post-operative neurological assessment of neurological function.

A major limitation of almost all CP studies is the poor discrimination of neurological recovery. It remains unclear whether the neurological recovery is promoted by CP surgery or represents the usual rehabilitation after the initial pathology (e.g., TBI, ischemia). These concerns can only be confirmed by a direct comparison of DC patients with and without CP surgery, which is not feasible due to ethical issues.

### Mortality and Survival

The long-term mortality rate (independent from CP-surgery) during follow up was 34%, whereas the surgery related mortality rate was only 0.49%. Long-term mortality rates are available for patients with DC after ischemia (15). A 5-year mortality rate of 31.1% after DC was reported, independent of the CP procedure. Kaplan-Meier analysis for patient survival showed similar results for our CP ischemia patients after 5 years. Gouello et al. (21) reported a 2-year mortality rate of 28.3% in their study on 60 patients treated with DC due to TBI. Despite the increased risk profile of patients with cerebral ischemia, Kaplan–Mayer survival analysis showed no differences in mortality for indication groups, most likely due to limited numbers of patients in the tumor and infection group.

### Quality of Life

Only very limited data on the QoL of patients after CP-surgery are currently available. It has been demonstrated that CP has a significant (*p* < 0.001) positive impact on QoL (17). Compared to our study, the authors evaluated the SF-36 at several time points following CP surgery whereas our study encompassed only one time point during the long term follow up (mean 91.9 month). Comparing the SF-36 sub-categories (after 24 month) with our study, worse results were observed in our patient cohort. However, this difference could be explained by the different indications for DC in both studies. Our study encompassed a heterogenous patient collective and did not focus only on DC due to TBI. Furthermore, our results showed that patients with DC and CP due to ischemia were more affected in their QoL (VAS 48.8 ± 21.8) than patients with other indications like TBI (VAS 67.6 ± 28.2). A systematic review had similar results with a mean QoL for ischemia patients of 46–67%, using VAS and questionnaires (26). Patient age differed significantly between the ischemia and TBI group. It can be hypothesized that higher age is associated with inferior possibilities of rehabilitation. Furthermore, permanent paralysis and decreased immobility seems to be more frequent in patients with ischemia than in TBI patients. In particular, the physical subcategories showed significantly worse results in ischemia patients compared to TBI patients. The effect of CP surgery on neurological recovery is unclear, as it is affected by several other factors associated with the underlying disease.

It is well-recognized that QoL represents an important outcome parameter after surgical procedures. Knowing how a (surgical) intervention affects a disease process can provide important information on the effectiveness and impact on the individual's own perception (27). Furthermore, QoL after neurocritical illness is associated with sequalae that are potentially amenable to medical treatment such as depression (28).

### Cosmetic Results

The majority of CP studies neglected the cosmetic results after DC/CP. Therefore, validated methods for evaluation of cosmetic results were only used in some case series or single center studies (3, 29–31). In our study more than a half of patients stated a very good or good cosmetic result and another 31% were satisfied. In total 13.5% of patients considered the cosmetic poor. Nevertheless, no patient was required to undergo revision surgery due to a poor cosmetic result. Poor cosmetic results have been reported in up to 50% of cases with a need of surgical revision in 1.5% of cases (30). However, direct comparison is difficult because cosmetic results were not assessed in a standardized manner. A major reason for a poor cosmetic result is the temporal muscle atrophy which occurred in two thirds of patients. Beside separate muscle preparation and fixation the only option to reduce this cosmetic problem is to manufacture special implants with an elevated curvature in the area of the muscle. Another option is a secondary subcutaneous fat implantation to fill the gap and elevate the curvature. Our statistical analysis did not identify factors associated with a good cosmetic outcome and no significant differences between autologous and patient specific implant (PSI).

### Limitations of this Study

The present study has several limitations. Patients were recruited at a single-center institution and analysis was performed in a retrospective manner. Follow-up data collection (neurological outcome, QoL, cosmetic result) was performed after DC/CP surgery which may have caused a selection bias. A considerable proportion of patients were not included in the present analysis. Furthermore, there was a large heterogeneity in patient characteristics between the different underlying conditions. Further studies (RCTs, registries) are necessary to prospectively analyze a pre- and post-operative as well as long-term outcome and QoL after DC/CP surgery. Two registry studies on CP have already been initiated in Europe, nevertheless results are still pending (32, 33).

## Conclusion

CP is a crucial step toward reintegration into daily life for patients. In the present study, we were able to show that in addition to the neurological outcome, especially the QoL and the cosmetic result of the CP procedures play important roles. Except for the differences in DC/CP indication, however, no other reason could be determined that may influence the QoL. Future studies should address these findings in specific patient populations and focus on more detailed evaluation and improvement of QoL. In addition, a standardized score for assessing the cosmetic results after CP should be introduced in order to enable a better comparability of further studies.

## Data Availability Statement

The raw data supporting the conclusions of this article will be made available by the authors, without undue reservation.

## Ethics Statement

The studies involving human participants were reviewed and approved by Ethics Committee of University of Heidelberg. The patients/participants provided their written informed consent to participate in this study.

## Author Contributions

HG initiated and performed the study and wrote the manuscript. JA were responsible for data collection and patient contact. AU read and corrected the manuscript. CB corrected the manuscript and supervised HG and JA during the study. All authors agree to be accountable for the content of the work.

## Conflict of Interest

The authors declare that the research was conducted in the absence of any commercial or financial relationships that could be construed as a potential conflict of interest.
